# Effect of Flat-Knitted Medical Compression Stockings on Venous Malformations

**DOI:** 10.3390/jcm12072723

**Published:** 2023-04-06

**Authors:** Yi Li, Antje Mükke, Ulrich Rother, Rolf Janka, Michael Uder, Werner Lang, Rafael Heiss

**Affiliations:** 1 Department of Vascular Surgery, University Hospital Erlangen, Friedrich-Alexander-Universität Erlangen-Nürnberg (FAU), Krankenhausstraße 12, D-91054 Erlangen, Germany; antje.muekke@uk-erlangen.de (A.M.); werner.lang@uk-erlangen.de (W.L.); 2 Institute of Radiology, University Hospital Erlangen, Friedrich-Alexander-Universität Erlangen-Nürnberg (FAU), Maximiliansplatz 1, D-91054 Erlangen, Germany; rolf.janka@uk-erlangen.de (R.J.); michael.uder@uk-erlangen.de (M.U.); rafael.heiss@uk-erlangen.de (R.H.)

**Keywords:** venous malformation, compression stockings, perometry, magnetic resonance imaging, T2-weighted imaging, quality of life

## Abstract

Venous malformations are one of the most common vascular anomalies. Our study aimed to investigate the effect of medical compression stockings of class I and II on the volume of venous malformations. Patients with venous malformations on upper or lower extremities were enrolled. They wore flat-knitted medical compression stockings of class I and II in a randomized order for four weeks each. Magnetic resonance imaging (MRI) and perometry were performed with and without wearing compression stockings. The 12-Item Short Form Survey (SF-12) questionnaire was performed before and after wearing compression stockings for four weeks each. A total of 18 patients completed the evaluations. Both compression classes showed a significant reduction of the volume of the venous malformations in the lesion itself based on MRI in comparison with baseline (both *p* < 0.001). Measurements taken with perometry did not reveal a significant difference in comparison to baseline (*p* = 0.09 and *p* = 0.22). The results of the SF-12 questionnaire demonstrated no significant differences before and after wearing the compression stockings of class I or class II for four weeks or between the two classes of compression therapy. Our results indicate that wearing medical compression stockings of both class I and class II significantly reduces the volume of venous malformation, without compromising the quality of life, while the effect of class II compression stockings on volume reduction was significantly better than that of class I.

## 1. Introduction

Venous malformation, defined as a low-flow lesion among vascular anomalies, is the most common form of congenital vascular malformation, which accounts for more than 50% of all vascular malformations, with a prevalence of around 1% [1,2,3,4]. Formed at birth, venous malformations grow with age and might lead to pain, edema or compression on the surrounding structures, which can lead to a reduced quality of life and the requirement of repetitive medical treatments in order to relieve the discomfort [2,5]. Despite the fact that medical compression stockings are commonly provided as a noninvasive therapy to relieve symptoms of venous and lymphatic disorders, prospective studies verifying the benefit of medical compression therapy in venous malformations are lacking [6,7]. The impact of compression therapy on the reduction of volume and its possible influence on the quality of life are left unclear.

For the diagnosis and evaluation of vascular malformation, MRI has been deemed as a reliable tool of investigation [7,8,9]. It is not uncommon that the clinical appearance is only the tip of the whole vascular lesion, which extends much deeper and more broadly underneath [10]. MRI provides valuable information including anatomical extension and involvement of the venous malformation, which sheds light on the planning for treatment and follow-up [11,12,13]. While MRI is able to measure the absolute volume of a venous malformation, perometry has been recognized as a reliable measurement for the whole limb volume, and both provide objective evaluation for volume reduction after compression therapy in our study [14,15,16].

Against this background, the aim of this prospective study is to evaluate the impact on volume reduction in venous malformation when wearing flat-knitted medical compression stockings of class I and class II by means of 3T MRI and perometry. In order to assess the influence of compression therapy on the quality of life, self-evaluated life quality was measured with the SF-12 questionnaire before and after the compression therapy. Furthermore, average daily wearing time and comfort of the medical compression stocking were assessed.

## 2. Materials and Methods

The study was approved by the ethics commission of the Friedrich-Alexander-Universität Erlangen-Nürnberg (124_17B) and registered in ClinicalTrials.gov with the registration number NCT04637997. All participants were informed of the benefits and risks of the investigation prior to signing an institutionally approved informed consent document to participate in the study.

**Figure 1:**
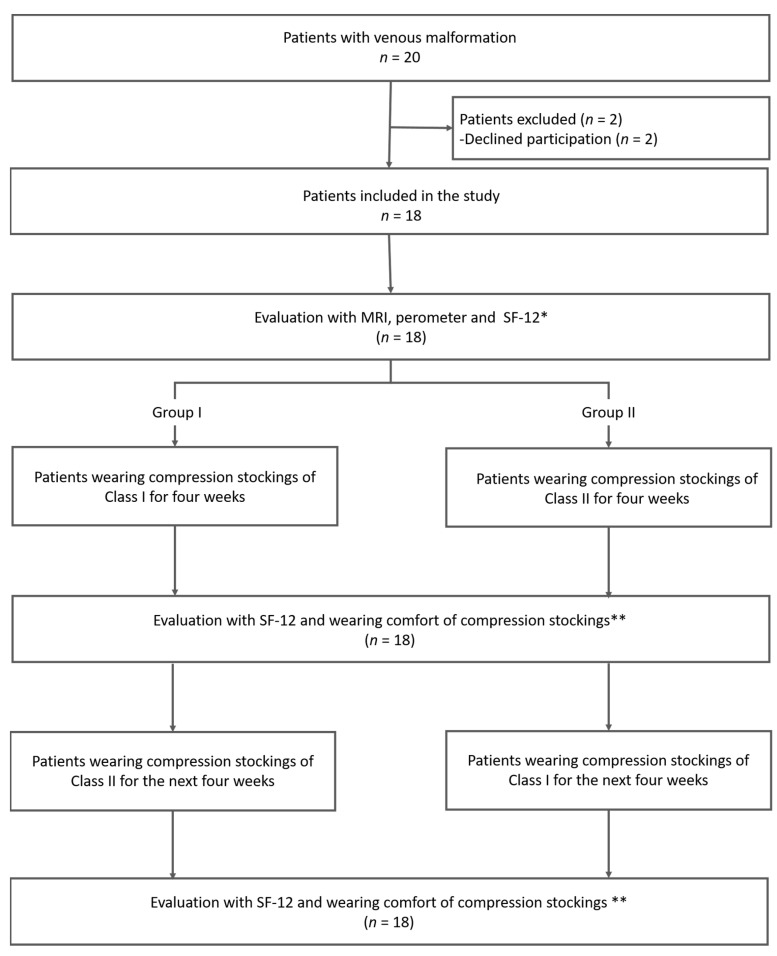
The study algorithm of patients included in the study. * First, MRI and perometry were performed as baseline measurements without wearing compression stockings. After that, medical compression stockings of class I and class II were offered in a randomized order and MRI and perometry were repeated for both classes of compression. SF-12 was exclusively assessed without wearing compression stockings. ** The compression stockings’ wearing comfort was evaluated with the German school grading system (a scale of 1–6, with 1 indicating very good wearing comfort and 6 insufficient wearing comfort).

Participants randomly and double-blinded received either class I (18–21 mmHg) compression stockings or class II (23–32 mmHg) compression stockings (flat-knitted medical compression stockings, mediven^®^ 550 arm/leg, medi, Bayreuth, Germany). After four weeks of therapy, an exchange of compression classes took place, so that all participants wore compression stockings of class I and II, each for a total of 4 weeks (Figure 1). The subjective assessment of the health-related quality of life was recorded using the SF-12 questionnaire, which was performed three times: before (baseline), after four weeks of compression stockings of class I or II and after the next four weeks after the exchange of compression classes. The total volume of the affected limb under different classes of compression stockings was measured with perometry, while the absolute volume of the vascular malformation was measured using MRI before and during wearing the compression stockings in the first visit (Figure 1).

### 2.1. Study Cohort

Twenty patients with known venous malformations in the upper or lower limb independent of prior or noninterventional therapy with epi- and/or subfascial localization of venous malformation independent of local extent (joint-crossing or not) were included, of which eighteen patients completed the evaluation. All patients were able to put on compression stockings independently or with the help of their parents.

### 2.2. Medical Device

CE-certified, RAL-compliant, flat-knitted medical compression stockings of class I and class II were made to measure. Flat-knitted medical compression stockings are subject to a special manufacturing process allowing increases and decreases in the number of meshes in the course, thus fitting even unique body shapes, a requirement often met within compression therapy of venous malformations. 

### 2.3. Magnetic Resonance Imaging

MRI of the venous malformation was performed without wearing compression stockings first. Immediately after the reference scan (baseline), a second and third scan when wearing a compression stocking were performed by the same technician. The different compression stockings were offered in a random order by two certificated medical supplier specialists. All researchers and patients involved were blinded to classes of the applied compression stockings. MRI contraindications were absent in all patients.

Imaging of the venous malformation was carried out at the Department of Radiology of the University Hospital Erlangen with a 3T MRI scanner (Magnetom Skyra Fit 3T, Siemens Healthineers, Erlangen, Germany) using a dedicated 32-channel spine coil and an 18-channel body coil (Siemens Healthineers).

An axial T2-weighted turbo inversion recovery magnitude (TIRM) sequence (total acquisition time, 3:31 min; inversion time, 260 ms; echo time, 69 ms; repetition time, 5120 ms; flip angle, 145°; resolution, 0.8 × 0.8 × 4.0 mm) and a coronal T2-weighted TIRM sequence (total acquisition time, 3:42 min; inversion time, 260 ms; echo time, 68 ms; repetition time, 5120 ms; flip angle, 180°; resolution, 0.9 × 0.9 × 4.0 mm) were applied to the venous malformation. Additionally, we performed a T1-weighted turbo spin-echo (TSE) sequence (total acquisition time, 3:01 min; echo time, 14 ms; repetition time, 582 ms; resolution, 0.7 × 0.7 × 5.0 mm) to depict the anatomy and morphology of the affected extremities.

### 2.4. Image Analysis

The MRI data were blinded and analyzed by one experienced, certificated cardio-vascular radiologist. Image analysis was performed using a professional image processing program (syngo.via VB10, Siemens Healthineers, Erlangen, Germany) to depict the extent of the venous malformation and to screen all patients for pathological abnormalities apart from the known venous malformations.

**Figure 2:**
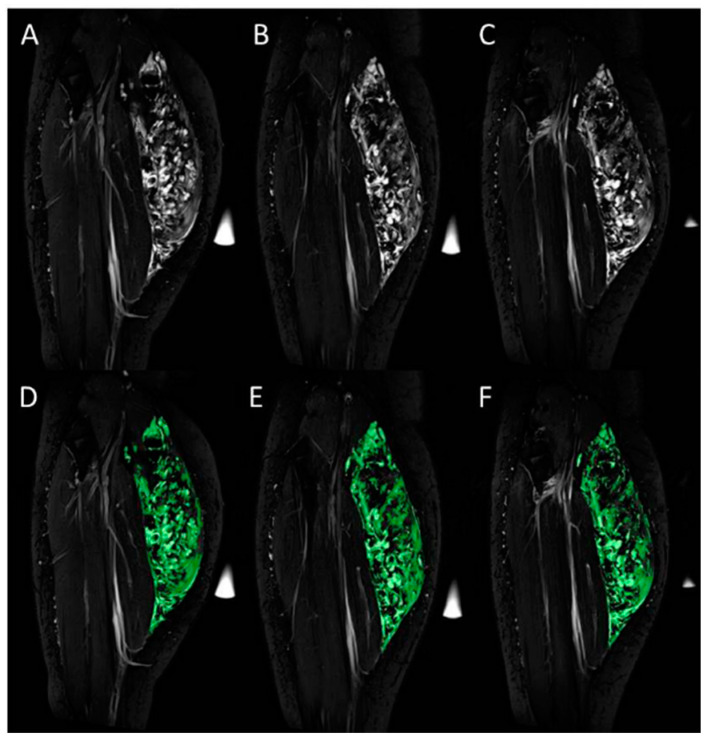
Volume of venous malformations was assessed in the T2-weighted TIRM images acquired without (**A**,**D**) and with wearing compression stockings of class I (**B**,**E**) and class II (**C**,**F**). Exemplary images before (**A**–**C**) and after color-coded manual segmentation of the venous malformation within the medial head of the gastrocnemius muscle are shown (**D**–**F**).

For calculating the volume of the venous malformation without and with wearing the compression stockings, T2-weighted TIRM signal intensities of the surrounding, not affected, muscle tissue were assessed by manually locating a region of interest. The threshold of the volume of the venous malformation was defined as the sum of T2-weighted TIRM signal intensity of the adjacent muscle tissue and the five-fold standard deviation. This threshold was used to determine the volume of the venous malformation via manual segmentation [17] (Figure 2). Commercially available software was used for the segmentation (Chimaera GmbH, Erlangen, Germany).

The test–retest reliability was evaluated by the same reader three months after the initial assessment by analyzing data sets of six randomly chosen patients. The results of the first investigation and the MRI data were blinded.

### 2.5. Perometry

Perometry is a noninvasive measurement of the volume of the limbs via an optoelectronic system using infrared beams, which has been broadly applied on the evaluation of lymphedema [15,18,19,20]. The diameter of the limb can be calculated to an accuracy of ±2.54 mm by the number of light diodes that are attenuated by the limb. The volume of the limb is then automatically calculated by the large number of vertical and horizontal diameter calculations of the elliptical or circular transverse profiles, which are determined at an interval of 3.1 mm. The data are passed directly to a computer for image display, where special software is used to determine the total volume. All patients underwent perometry measurement before and during application of compression stockings (class I and II), all within one single visit. 

### 2.6. Twelve-Item Short Form Survey Questionnaire

Subjective assessment of health-related quality of life was assessed using the SF-12 questionnaire. The SF-12 is a cross-disease measurement instrument for assessing the health-related quality of life of patients which is composed of 12 items [21]. It is designed as an instrument for recording therapy success by means of subjective assessment of health-related quality of life by patient groups. The SF-12 covers eight dimensions of subjective health: physical functioning, physical role functioning, pain, general health perception, vitality, social functioning, emotional role functioning and psychological well-being. The questionnaire aims to quantify the quality of life in both physical and mental aspects [22]. The questionnaire was completed by all patients before, after four weeks of wearing the compression stockings class I or II and after the second four weeks of wearing stockings with the different compression class.

### 2.7. Average Daily Wearing Protocol and Wearing Comfort of Compression Stockings

Study participants were asked to daily document wearing time in a personal logbook. The wearing comfort of compression stockings was assessed by a questionnaire. Since children were also enrolled in the study, the German school grading system was used for the evaluation.

### 2.8. Statistical Analysis

Although the experiment was conducted with a cross-over design, carryover or period effects were not expected due to the nature of the treatments. Therefore, differences between the treatments and baseline measurements were evaluated using Wilcoxon signed rank tests. Intraobserver reliability was evaluated by using the intraclass correlation coefficient (ICC). Statistical significance was defined as a *p*-value less than or equal to 0.05. All results were calculated using R version 3.5.1 (R Foundation for Statistical Computing, Vienna, Austria).

## 3. Results

### 3.1. Patient Characteristics

The characteristics of the 18 patients are provided in Table 1. The mean age was 25.7 (9–62) years old. Five patients (28%) had venous malformations on the upper extremities, while the other thirteen patients (72%) on the lower extremities. One patient had chronic kidney disease and hypertension. None of the patients had diabetes. Two patients (11%) received long-term anticoagulants for thrombotic prophylaxis. By the time of recruiting, ten patients (56%) had previously received sclerotherapy.

### 3.2. Reduction of the Volume after Wearing Medical Compression Stockings

In comparison with the baseline measurements (without compression stockings), both compression classes showed a significant reduction of the volume of the venous malformation in the MRI (both *p* < 0.001). By comparing compression classes I and II, class II showed a significantly higher volume reduction (related to baseline) than class I (*p* = 0.04). Measurements of the whole limb’s volume taken with perometry did not reveal a significant difference in comparison to baseline values (*p* = 0.09 and *p* = 0.22) (Table 2 and Table 3). The volume of venous malformations or affected limbs of each patient at baseline and during wearing compression stockings of class I/II was listed in Table 4.

### 3.3. Influence of Compression Stockings on the Quality of Life

**Figure 3:**
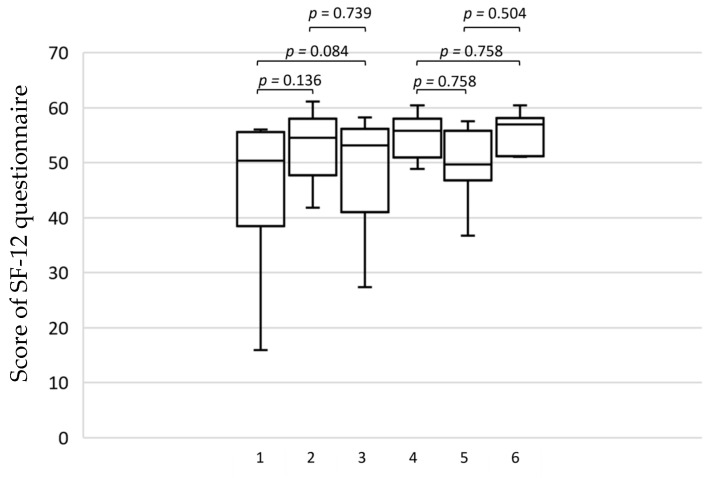
Median and range of the physical and mental health between compression class I and class II assessed with SF-12 questionnaire. 1: physical score at baseline, 2: physical score after wearing compression stocking of class I for four weeks, 3: physical score after wearing compression stocking of class II for four weeks, 4: mental score at baseline, 5: mental score after wearing compression stocking of class I for four weeks, 6: mental score after wearing compression stocking of class II for four weeks.

The results of the SF-12 questionnaire indicated that there were no significant differences either physically or mentally before and after wearing the compression stockings of class I or class II for four weeks (*p* = 0.14 and 0.08 in physical score; *p* = 0.76 and 0.76 in mental score) or between the two classes of compression therapy (*p* = 0.74 in physical score and *p* = 0.50 in mental score) (Figure 3).

### 3.4. Average Daily Wearing Protocol and the Wearing Comfort of Compression Stockings

Average daily wearing time documented by the patients wearing class I compression stockings was 12.22 h per day (SD = 3.78), while that by the patients wearing class II was 12.11 h (SD = 4.10) (Table 5).

The overall wearing comfort was very satisfying, with a mean of 1.61 (SD = 0.60) for medical compression stockings of class I and a mean of 2.00 (SD = 0.86) for medical compression stockings of class II (on a scale of 1–6, with 1 indicating very good wearing comfort and 6 insufficient wearing comfort) (Table 6).

No study-related adverse events occurred during the entire study.

## 4. Discussion

To our knowledge, this is the first study assessing compression stockings in patients with venous malformations by objectively evaluating the change in the volume under medical compression therapy using 3T MRI and perometry. In addition, a self-reporting questionnaire was used to evaluate the impact of compression therapy on daily life. Our results indicate that wearing compression stockings significantly reduces the volume of the venous malformation without compromising the quality of life, independent of the compression class (class I or class II) applied.

Compression therapy is broadly prescribed as the first-line conservative therapy in terms of ulcer wound healing, pain relieving, prevention of thrombosis and lymphedema. There is plenty of literature focusing on both therapeutic and preventive effects of compression therapy, yet several issues remain controversial [17,23,24,25,26]. Rabe et al. examined the evidence level of indications for medical compression stockings in various venous and lymphatic diseases in a systematic review and supported their usage in patients with chronic venous diseases for the alleviation of symptoms and the improvement in quality of life [27]. However, in this systematic review, venous diseases were merely classified into acute or chronic, and further detailed classification such as the flow rate is missing. In a systematic review published by Langbroek et al., the absence of high-quality evidence supporting the application of compression therapy in low-flow vascular malformations was pointed out [6]. C.R. Lattimer published a prospective study examining the hemodynamic performance of compression stockings in venous and lymphatic diseases, which showed that the improvement varies among healthy groups, patients with varicose veins, post-thrombotic syndrome and lymphedema [28].

Against this background, we carried out a double-blinded, randomized trial that aimed to evaluate the efficacy of compression therapy in both an objective and subjective manner. Both MRI and perometry are well-established, reliable methods to measure the change in volume of soft tissue [14,15,16,18,20]. The measurement using MRI showed a significant reduction in volume during wearing of compression stockings for class I and class II. The assessment using perometry did not show significant changes between baseline and compression class I or class II. A reasonable explanation for the controversial results may be that MRI assessed the volume of the venous lesion itself, while the whole volume of the extremity was measured by perometry, which naturally relativizes the effect. The mean reduction of the volume of the evaluated venous malformations measured with MRI was 16.4 mL in class I and 23.7 mL in class II. These volume changes are smaller than previously suggested minimally detectable alterations using perometry [29]. Therefore, MRI seems to be superior for the evaluation of changes of the volume of venous malformations compared to perometry.

Furthermore, the results from SF-12 indicated that there was no significant difference in regard to general health-related quality of life between the two classes of compression stockings. As the effects on the reduction in volume by class I and class II are both statistically significant, without further compromising the quality of life, both classes of compression stockings could be deemed as suitable choices for patients with venous malformations. Compression stockings are expected to be able to relieve the symptoms of venous malformation and further improve the quality of life in patients, which was not observed in our study. A reasonable explanation would be that the quality of life at baseline of our patients was only slightly affected by the venous malformations, therefore no significant effect was observed. However, considering that the volume of the lesions measured by MRI is significantly reduced during wearing compression stockings, it might be helpful for patients with more severe, malformation-related symptoms. Further studies are required to validate this assumption.

Patients’ compliance and adherence are decisive for the success of compression therapy. In the present study, the wearing comfort of compression therapy was almost exclusively rated as good or very good, and the average daily wearing time was 12.22 h per day for class I compression stockings and 12.11 h per day for class II compression stockings, demonstrating highly satisfying adherence. Given the fact that medical compression therapy often is a conservative therapeutic option already used in childhood, our study’s results imply that both class I and class II could be taken into account as a first approach for beginners/children, without compromising the medical effect and compliance.

There are some limitations in our study. First, the number of participants included is small. Second, our data are representative of the applied study setting in which the venous malformations are evaluated without and with two different types of compression stocking over a period of three months, which should be considered cautiously in a different setting. A third limitation is the fact that incorrect use or a lack of compliance might be possible. However, considering that this setting is similar to clinical practice, the result should be deemed as reliable and suitable for further application.

## 5. Conclusions

Our results indicate that wearing medical compression stockings of both class I and class II significantly reduces the volume of venous malformation, without compromising the quality of life, while the effect of class II compression stockings on volume reduction is significantly better than that of class I. In addition, high compliance and satisfying reports regarding wearing comfort of compression stockings were observed. By evaluating the impact of compression therapy in both an objective and subjective manner, our study provides insights into the therapeutic strategies in venous malformations. Both class I and class II of flat-knitted medical compression stockings can be recommended as suitable noninvasive therapy choices for patients with venous malformations.

## Figures and Tables

**Table 1:** Patients’ characteristics.

Patients’ Characteristics (*n* = 18)	
Age (median, minimal–maximal)	18.5 (9–62)
Male (number, %)	6 (33.3%)
Affected region	
Upper extremities	5 (27.8%)
Lower extremities	13 (72.2%)
CKD	1 (5.9%)
DM	0 (0%)
HTN	1 (5.9%)
Long-term anticoagulants	2 (11.1%)
Previous sclerotherapy	10 (55.6%)

CKD: chronic kidney disease; DM: diabetes mellitus; HTN: hypertension.

**Table 2:** Volume of venous malformations assessed with MRI and volume of the affected extremity assessed with perometry. Measurements were performed at baseline (without compression) and during wearing compression stockings of class I or class II. *p*-values represent the comparison of class I and class II to baseline values.

	Baseline	Class I	*p*-Value	Class II	*p*-Value
MRI, median (minimal–maximal)	77.7 (2.8–394.6)	58.4 (2.5–343.1)	<0.001	53.9 (2.4–371.5)	<0.001
Perometry, median (minimal–maximal)	5804.5 (1467.0–10514.0)	5774.5 (1467.0–9348.0)	0.09	5733.5 (1521.0–10788.0)	0.22

**Table 3:** Mean and range for the absolute reduction of the volume (ml) of venous malformations in MRI.

	Class I	Class II	*p*-Value
Reduction in volume in MRI, mean (SD)	16.4 (23.90)	23.7 (21.20)	0.04

**Table 4:** The volume of venous malformations/affected limbs of each patient at baseline and during wearing compression stockings of class I/II.

Patients	Volume of Venous Malformations Assessed by MRI (mL)			Volume of Affected Limb Measured by Perometer (mL)		
	Baseline	Class I	Class II	Baseline	Class I	Class II
1	219.60	209.12	168.14	3822.00	4110.00	3893.00
2	34.72	36.40	34.69	3778.00	3916.00	4096.00
3	275.00	259.92	246.46	10514.00	9342.00	10788.00
4	56.95	54.97	54.27	4991.00	5013.00	4992.00
5	118.41	115.80	108.84	8747.00	8579.00	8644.00
6	10.42	5.70	7.10	2017.00	2086.00	2042.00
7	19.01	10.52	16.07	3962.00	4132.00	4131.00
8	3.20	2.49	2.43	6618.00	6536.00	6475.00
9	196.60	161.54	165.23	6776.00	6800.00	6662.00
10	307.23	222.11	247.23	9589.00	9240.00	9250.00
11	82.56	61.83	53.50	1467.00	1467.00	1521.00
12	2.84	2.50	2.58	7592.00	7518.00	7504.00
13	72.77	37.91	40.74	4491.00	4549.00	4557.00
14	7.88	8.75	7.77	4059.00	3854.00	3910.00
15	14.03	12.49	13.33	9680.00	9348.00	9491.00
16	127.00	98.65	72.05	9494.00	9302.00	9244.00
17	112.57	63.40	70.29	3254.00	3175.00	3084.00
18	394.63	343.10	371.47	7727.00	7040.00	7009.00

**Table 5:** Mean and range of wearing time (hours per day) of the medical device.

	Class I	Class II
Daily wearing time of compression stockings, mean (SD)	12.22 (3.78)	12.11 (4.10)

**Table 6:** Mean and SD of wearing comfort of the medical device (with 1 indicating very good and 6 insufficient wearing comfort).

	Class I	Class II
Evaluation of compression stockings’ wearing comfort, mean (SD)	1.61 (0.60)	2.00 (0.86)

## Data Availability

Data presented in this study are available upon request.
